# Acne, Its Etiology, Pathology and Treatment

**Published:** 1885-12

**Authors:** 


ACNE—ITS ETIOLOGY, PATHOLOGY, AND TREATMENT. By L. Duncan Bulkley, A. M., M. D. New York: C. P. Putnam's Sons. 8vo., pp., 280. 1885.

The author of this work needs no introduction to the profession. He is known by those excellent works on dermatological subjects which have appeared from time to time from his prolific pen. The book before us is a full treatise on the nature and treatment of one of the most obstinate and troublesome diseases of the skin, and anything new that is offered bearing on the treatment is always eagerly received.

Although the author gives a full and clear exposition of the nature of acne, he offers little in the way of treatment that has not already been suggested. Still the chapters devoted to treatment are of great worth, in that the methods of treatment are not merely indicated, but are given with those details that are necessary to a successful handling of this affection. Special attention is directed to constitutional disturbances, especially functional derangements of the alimentary canal. We are glad to notice that more attention is being paid to the influence of internal disorders upon diseases of the skin, and that such influences are counteracted by proper internal medication. The Vienna idea, still prevalent to a degree, that all skin affections, except syphilis, are to be treated locally, is fast dying out.

The directions as to diet are clear and significant, and the appended formulary contains many valuable combinations of remedies. The complete bibliography at the end of the book will be appreciated by those who look more specially into the subject.

